# Endoscopic immediate hemostasis strategy for palatal hematoma induced by linear endoscopic ultrasound

**DOI:** 10.1055/a-2729-2804

**Published:** 2025-11-21

**Authors:** Ye Han, Sheng Wang, Siyu Sun

**Affiliations:** 1 85024 Department of Gastroenterology, Shengjing Hospital of China Medical University, Shenyang, China

Linear echoendoscopes have the ultrasound transducer located at the tip, which make them more rigid than standard endoscopes. Although complications are rare 1 2 , endoscopic ultrasonography (EUS) carries inherent risks, particularly hemorrhage 3 . We report a rare case of a palatal hematoma during diagnostic EUS. The bleeding was subsequently controlled by using biopsy forceps under endoscopic vision.

A 61-year-old male was referred to our department for an EUS diagnosis under general anesthesia. During the examination, fresh blood was observed at the corner of the patientʼs mouth. The gastroscope was immediately replaced ( Video 1 ), and endoscopic visualization revealed hematoma formation with active bleeding in the upper palate ( Fig. 1 ). Under non-intubated general anesthesia, the patient was at risk of aspiration, and hemostasis was urgent. Under gastroscopy, fresh blood was rapidly aspirated from the mouth, and the bleeding point was accurately located. First, we used the gastroscope itself to compress the hematoma and reduce its tension. And then, the biopsy forceps was inserted to precisely clamp the bleeding vessel ( Fig. 2 ). After four rounds of biopsy forceps compression, each lasting 2–3 minutes, hemostasis was achieved ( Fig. 3 ). When the patient woke up, there was no bleeding in the mouth, and he had no discomfort when drinking water 2 hours later. The patient did not experience bleeding symptoms again during follow-up on the second day.

The video shows a palatal hematoma formation and bleeding during diagnostic EUS. The bleeding was subsequently controlled by using biopsy forceps under endoscopic vision.Video 1

**Fig. 1:**
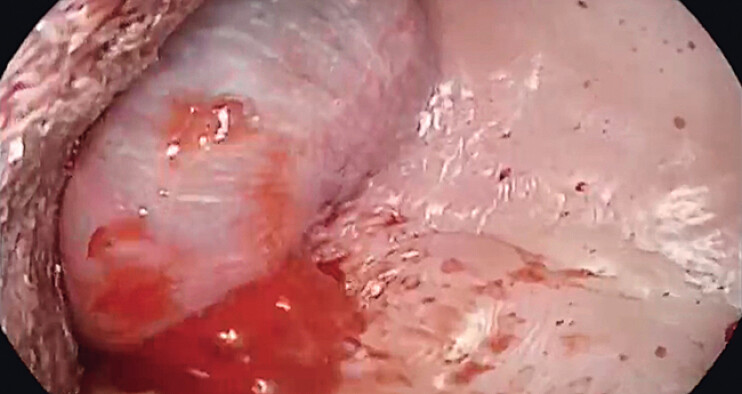
Endoscopic visualization revealed hematoma formation with active bleeding in the upper palate.

**Fig. 2:**
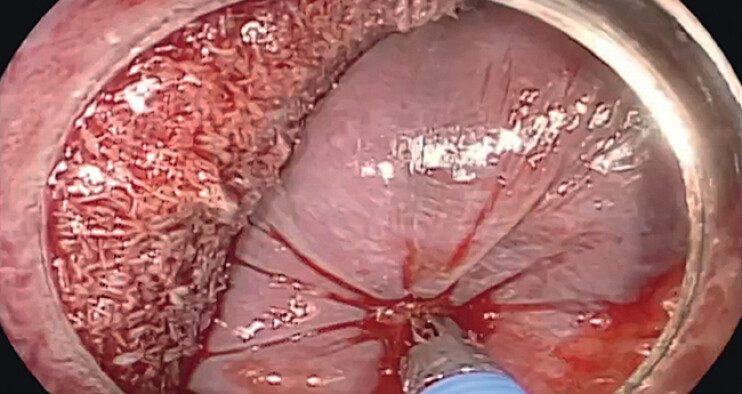
The biopsy forceps was inserted to precisely clamp the bleeding vessel.

**Fig. 3:**
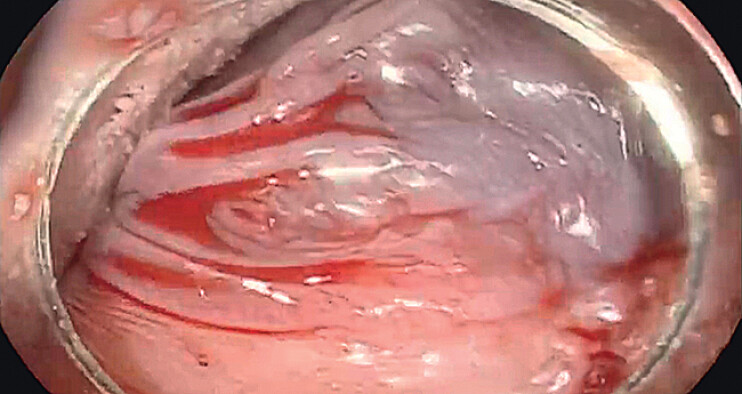
Bleeding clearly stopped after releasing the biopsy forceps, with no further expansion of the hematoma.

Standard treatments for oral bleeding include gauze packing, surgical suturing, and hemostatic sponges 4 , with no reported cases of endoscopic hemostasis. Conventional endoscopic hemostasis methods involve hemostatic clips and electrocoagulation, which cause significant postoperative discomfort and impair short-term quality of life. In our case, endoscopic hemostasis using biopsy forceps compression achieved immediate, effective, and precise hemostasis. No adverse events such as coughing or aspiration occurred. This treatment strategy has great clinical practicality, particularly during EUS procedures.

Endoscopy_UCTN_Code_CPL_1AH_2AC

## Notes

Endoscopy E-Videos https://eref.thieme.de/e-videos
*E-Videos* is an open access online section of the journal *Endoscopy* , reporting on interesting cases and new techniques in gastroenterological endoscopy. All papers include a high-quality video and are published with a Creative Commons CC-BY license. Endoscopy E-Videos qualify for HINARI discounts and waivers and eligibility is automatically checked during the submission process. We grant 100% waivers to articles whose corresponding authors are based in Group A countries and 50% waivers to those who are based in Group B countries as classified by Research4Life (see: https://www.research4life.org/access/eligibility/ ). This section has its own submission website at https://mc.manuscriptcentral.com/e-videos .
